# Diversity of Rumen Bacteria in Canadian Cervids

**DOI:** 10.1371/journal.pone.0089682

**Published:** 2014-02-27

**Authors:** Robert J. Gruninger, Christoph W. Sensen, Timothy A. McAllister, Robert J. Forster

**Affiliations:** 1 Lethbridge Research Center, Agriculture and Agri-Food Canada, Lethbridge, AB, Canada; 2 Faculty of Medicine, Department of Biochemistry and Molecular Biology, University of Calgary, Calgary, AB, Canada; Field Museum of Natural History, United States of America

## Abstract

Interest in the bacteria responsible for the breakdown of lignocellulosic feedstuffs within the rumen has increased due to their potential utility in industrial applications. To date, most studies have focused on bacteria from domesticated ruminants. We have expanded the knowledge of the microbial ecology of ruminants by examining the bacterial populations found in the rumen of non-domesticated ruminants found in Canada. Next-generation sequencing of 16S rDNA was employed to characterize the liquid and solid-associated bacterial communities in the rumen of elk (*Cervus canadensis*), and white tailed deer (*Odocoileus virginianus*). Despite variability in the microbial populations between animals, principle component and weighted UniFrac analysis indicated that bacterial communities in the rumen of elk and white tail deer are distinct. Populations clustered according to individual host animal and not the association with liquid or solid phase of the rumen contents. In all instances, *Bacteroidetes* and *Firmicutes* were the dominant bacterial phyla, although the relative abundance of these differed among ruminant species and between phases of rumen digesta, respectively. In the elk samples *Bacteroidetes* were more predominant in the liquid phase whereas *Firmicutes* was the most prevalent phyla in the solid digesta (P = 1×10^−5^). There were also statistically significant differences in the abundance of OTUs classified as *Fibrobacteres* (P = 5×10^−3^) and *Spirochaetes* (P = 3×10^−4^) in the solid digesta of the elk samples. We identified a number of OTUs that were classified as phylotypes not previously observed in the rumen environment. Our results suggest that although the bacterial diversity in wild North American ruminants shows overall similarities to domesticated ruminants, we observed a number of OTUs not previously described. Previous studies primarily focusing on domesticated ruminants do not fully represent the microbial diversity of the rumen and studies focusing on non-domesticated ruminants should be expanded.

## Introduction

Cellulose is a principal component of plant cell walls and the complete hydrolysis of this polymer requires the synergistic activity of a wide range of carbohydrate degrading enzymes. One of the best-characterized systems capable of effectively breaking down complex cellulolytic biomass is the rumen microbial community. In recent years, interest in plant cell-wall-degrading microbes and enzymes has increased due to the numerous potential industrial applications of these organisms and the proteins they express. In ruminants, digestion of the ingested plant biomass takes place under anaerobic conditions in the rumen. This anaerobic digestion chamber is inhabited by a diverse community of bacteria, archaea, protozoa and fungi that maintain a symbiotic relationship with the host, with bacteria playing the primary role in biomass degradation. These bacteria produce an array of enzymes that break down the lignocellulosic material. The resulting sugars are fermented into volatile fatty acids and used by the ruminant host for energy [1]. Considerable effort has been put forth into understanding the complex biology of this microbial ecosystem, including the application of metagenomics [2], metatranscriptomics [3], and genomic studies of major polysaccharide-degrading bacteria, as well as characterization of the enzymes they produce [4].

The rumen microbiome has been extensively studied, as the composition of this community can have a great impact on rumen function/dysfunction [4], [5], [6]. Until recently, the rumen microbiome was primarily studied using culture-based or classical molecular techniques (*i.e*., DGGE and ribosomal RNA clone libraries, respectively). Recent advances in next-generation sequencing have enhanced our ability to study this extremely complex environment. A recent examination of rumen microbe species in Genbank suggests that despite the extensive efforts that have gone into examining the microbial diversity of the rumen, there is still a great deal that we have yet to find out about this extraordinary environment [7]. To date, most studies have focused on domesticated ruminants, however, it is well known that the composition of the rumen microbial community varies depending on diet and ruminant species [7], [8], [9], [10]. There has been some effort to examine the microbial diversity of wild ruminants, but these studies have primarily examined a small number of clones using traditional 16S rDNA cloning techniques [8], [11], [12]. More recently, next generation sequencing approaches have been used to examine the bacterial diversity in the rumen of bovine [13], [14] the North American moose [15] and the Norwegian reindeer [16]. The diets of domesticated ruminants usually consist of high-quality forages or concentrates (*e.g*. hays, silages, or grain concentrates), whereas the diets of wild ruminants is more varied, depending on the nature of the browse and forage available for consumption at a given point in time within the environment. Given the different feeding strategies that are utilized by wild and domesticated ruminants, one would expect that the microbial populations in these hosts should be distinct. This is supported by a recent transcriptomic analysis of the rumen of musk-ox, which found a diverse array of genes coding for highly novel fibrolytic enzymes expressed in these animals [3] as well as the metagenomic analysis of the rumen microbiome of Norwegian reindeer [16].

We have conducted a study to examine the diversity of the rumen bacterial communities found in a number of wild Canadian ruminants using next-generation sequencing. Pyrosequencing of the V1-V3 region of the rRNA gene has been used to identify the prokaryotic diversity found in elk (*Cervus canadensis*), and white tailed deer (*Odocoileus virginianus*). Additionally, we have examined the differences between the bacterial communities associated with the solid and liquid fractions of rumen contents in these animals.

## Materials and methods

### Ethics Statement

Rumen samples from white tail deer were obtained, with permission, from animals harvested by licensed hunters during the open rifle fall hunting season in Sothern Alberta, during the course of their regular hunting activities. Rumen samples from elk were collected with permission at a licensed abattoir (Ft. Macleod, Alberta) immediately after animals were harvested. No animals were killed specifically for this study.

### Sample collection

Samples of rumen contents were removed from the rumen immediately after death and transported in an airtight container to a field lab within 30 min. Rumen samples from elk were collected at an abattoir immediately after animals were harvested. The elk were non-domesticated farmed animals that grazed on native prairie forage on farms in southern Alberta and southwestern Saskatchewan. Total rumen contents from elk (n = 15) and deer (n = 3) were transferred to a heavy walled 250 mL beaker and the solid and liquid phases were separated using a Bodum coffee filter plunger (http://www.bodum.com/ca/en-us/). Rumen contents were separated to evaluate differences in the microbial community associated with the solid and liquid digesta. Subsamples (5 mL) of liquid phase and ∼5 g of rumen solids were collected and frozen in liquid nitrogen and subsequently stored at −80°C.

### DNA extraction

Frozen samples were ground in a Retsch RM 100 Mortar Grinder with the addition of 30 mL of 100 mM Tris-HCl pH 8.0, 500 mM EDTA pH 8.0, 1.5 M NaCl, 1 mg/mL Proteinase K in the presence of liquid nitrogen for 5 min. Following grinding, the samples were incubated at 50°C for 40 min, combined with 3 mL 2% SDS and incubated at 65°C for another 45 min. The lysate was centrifuged at 19,200×g for 10 min at room temperature to pellet debris. The lysate supernatant was combined 1∶1 (v/v) with 65°C 2% agarose, then poured into 90 mm square petri plates. The agarose containing the embedded DNA was equilibrated 3 times over 24 h against 30 volumes of TE (10 mM Tris pH 8.0, 1 mM EDTA pH 8.0) buffer and subsequently stored at 4°C. Large molecular weight DNA was eluted from the agarose using the “Freeze squeeze” method [17]. The DNA concentration was determined using the Quant-iT PicoGreen dsDNA assay kit, according to the manufacturers protocol (Life Technologies). Sequencing of the 16S rRNA genes was performed by Molecular Research LP, where pyrosequencing was carried out using the bTEFAP FLX massively parallel method [18]. A 100 ng DNA aliquot was used as a template in a 50 µL PCR reaction. The 16S rRNA gene universal bacterial primers 27F 5′-AGAGTTTGATCMTGGCTCAG-3′ [19] and 519R 5′-AGRGTTTGATCMTGGCTCAG-3′ [20] were used to obtain a 450 bp amplicon. The pyrosequencing targeted the V1-V3 hypervariable region of the 16s rRNA, using the procedure of Dowd et al., 2008. The metadata and sequence reads are available at the European Nucleotide Archive (http://www.ebi.ac.uk/ena) under study accession number PRJEB4222 and run accession numbers ERR318187-ERR318224.

### Sequence analysis

Mothur v1.25 [21] was used for sequence analysis, OTU detection, taxonomic assignment and phylogenetic analysis. Weighted UniFrac calculations [22] and principle component analysis, using the Jaccard index, were carried out with Mothur to compare bacterial populations among different samples. The sequence data was first subjected to stringent quality control. This involved the use of pyronoise/flowgram noise reduction within Mothur, removal of all sequences shorter than 200 bp, sequences with more than 1 mismatch in the barcode region, 2 mismatches in the primer sequence or those that had homopolymers >9. The remaining sequences were aligned using the Silva bacterial database [23], which contained additional 16s sequences from an in-house database of rumen bacteria. After the alignment, the ends of the sequence were optimized using the optimize = end command in Mothur. Chimeric sequences were detected and removed using the sequence collection (UCHIME) as its own reference dataset [24]. Sequences were then subsampled to obtain a uniform number of sequences per sample for all subsequent analyses. A distance matrix was constructed using with Mothur at phylogenetic distances of 0.03 (species), 0.07 (genus) and 0.25 (phylum), respectively, to define OTUs. Mothur was also used to calculate sequence coverage, species diversity using inverse Simpson and Shannon-Weiner indices (Table 1), to create a cladogram based on differences in microbial communities using the Jaccard index, and to define the core microbiome in samples. Student t-tests were carried out to examine the significance of differences in the abundance of OTUs in the samples. Differences with a P-value <0.05 were determined to be statistically significant. Taxonomic identity of the OTUs belonging to the core microbiome was identified using ARB, with the same Silva bacterial database used for the sequence alignment.

**Table 1:** Sequence coverage, number of OTUs and richness of rumen samples included in this study at 93% similarity.

	Coverage (%)	Observed OTUs	Inverse Simpson	Shannon
White Tailed Deer				
**WTD1S**	**86.7**	**685**	**63.8**	**5.32**
*WTD1L*	90.0	531	30.0	4.62
**WTD2S**	**84.0**	**859**	**86.1**	**5.80**
*WTD2L*	85.3	720	36.6	5.25
**WTD3S**	**84.3**	**818**	**111.0**	**5.75**
*WTD3L*	85.5	823	155.2	5.91

Elk				
**E7S**	**77.6**	**1099**	**219.9**	**6.32**
*E7L*	80.2	994	171.6	6.13
**E9S**	**76.2**	**1138**	**204.1**	**6.35**
*E9L*	78.8	1038	149.0	6.19
**E70S**	**82.4**	**847**	**86.8**	**5.69**
*E70L*	84.5	766	145.7	5.76
**E13S**	**78.5**	**1076**	**158.2**	**6.22**
*E13L*	83.3	828	89.5	5.70
**E14S**	**73.0**	**1227**	**311.0**	**6.52**
*E14L*	74.5	1159	302.6	6.42
**E17S**	**77.5**	**1114**	**229.2**	**6.42**
*E17L*	77.3	1105	241.7	6.35
**E20S**	**77.4**	**1064**	**201.6**	**6.21**
*E20L*	75.0	1177	96.2	6.30
**E22S**	**81.4**	**931**	**127.3**	**5.93**
*E22L*	79.8	971	143.8	5.93
**E26S**	**89.9**	**538**	**30.4**	**4.73**
*E26L*	88.2	639	52.4	5.19
**E29S**	**87.6**	**712**	**96.4**	**5.57**
*E29L*	88.2	657	75.6	5.43
**E32S**	**82.8**	**849**	**85.8**	**5.70**
*E32L*	81.6	936	177.1	5.69
**E57S**	**76.9**	**1130**	**290.8**	**6.41**
*E57L*	76.2	1120	215.6	6.33
**E60S**	**81.7**	**893**	**96.0**	**5.78**
*E60L*	87.7	701	135.4	5.78
**E62S**	**82.8**	**896**	**167.1**	**6.02**
*E62L*	84.8	806	74.1	5.73
**E64S**	**81.1**	**920**	**137.7**	**5.97**
*E64L*	82.7	924	171.0	6.07

Bold samples correspond to rumen solids and italic samples correspond to rumen liquids. The moose sample was mixed and contained both rumen liquid and solid. WTD – white tail deer and E – elk. Samples ending with S or L correspond to solid or liquid phases, respectively.

## Results

### Pyrosequencing of elk and white tailed deer rumen samples

Pyro-sequencing of the V1-V3 region of the 16s rDNA gene in all of the elk and white tailed deer samples resulted in a total of 178,912 sequences being identified, 81,266 of which were unique. After applying quality control, 158,513 total and 60,399 unique sequences were retained. Sequences were subsampled (n = 2,862) to ensure a consistent and equal number of sequences from each sample were used in all comparisons and calculations. Representative rarefaction curves for the samples analyzed in this study can be found in Figure S1. The elk samples have a higher number of OTUs in both rumen phases compared to deer (Student t-test, P = 0.006) and species richness as estimated by both Inverse Simpson and Shannon indices was higher in elk compared to deer (P = 0.009 and 0.04, respectively). The sequence coverage, observed OTUs, and species richness, as estimated using the Inverse Simpson and Shannon indices, are shown in Table 1.

### Bacterial community composition in wild ruminants

**Figure 1:**
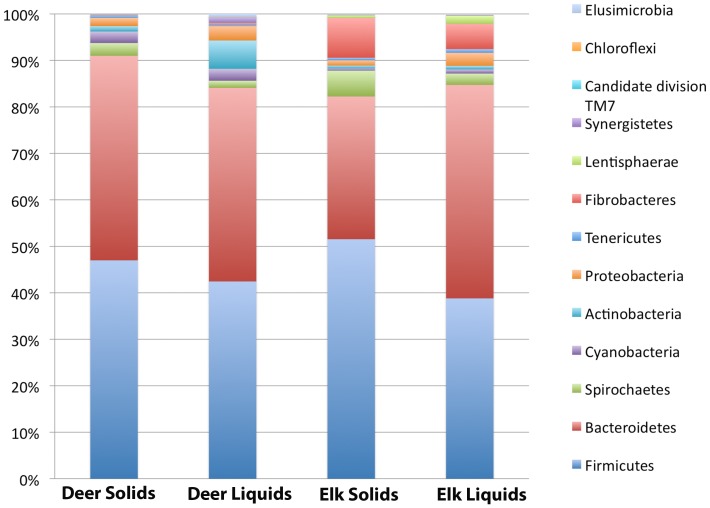
Phylum level comparison of rumen microbiome associated with solid and liquid rumen phase from white tailed deer (n = 3) and elk (n = 15). Values shown represent the percent of sequences assigned to a particular phylum averaged over all samples of the same type.

As in all other rumen environments examined to date, Bacteroidetes and Firmicutes were the predominant bacterial phyla in all samples, however, the proportion of sequences in each phylum varied in all samples examined (Fig. 1). At the class level, Clostridia and Bacteroidia were the most prevalent in both elk and deer. We observed a significant number of sequences belonging to Fibrobacteria in all elk samples, while these were almost completely absent in deer samples (P = <1×10^−7^). In total, 83 orders, 141 families, and 327 bacterial genera were identified when the samples across all animals.

*Prevotella* made up 19.8% and 11.6% of the sequences in the solid phase and 27.5% and 18.2% of the sequences in the liquid phase of white tailed deer and elk, respectively. This indicates that *Prevotella* is more abundant in the liquid phase of the rumen compared to the solid phase in both white tail deer and elk (P = 0.001). Sequences assigned to the uncultured rumen bacterial cluster RFN8-YE57 were observed in all wild ruminants in this study, but were most abundant in elk (14.1%) and were more prevalent in rumen solids than liquids (P = 0.005).

*Quinella ovalis* made up 5.4% of the sequences in white tailed deer, but only 0.1% in elk. The abundance of *Quinella* sequences also varied between the solid and liquid phase and were approximately 2-fold higher in the liquid phase of the deer samples, however this trend was not found to be statistically significant (P = 0.48). Another member of the *Veillonellaceae* family, a species related to the genus *Anaeromusa*, was identified at similar levels in the solid and liquid phase rumen contents from white tailed deer (2.1%) but was almost absent from elk (<0.1%). An OTU that was identified as being similar to the genus *Saccharofermentans* and clustered with a number of uncultured rumen bacteria, was also abundant in the elk samples corresponding to 2.1 and 2.8% of the sequences in the liquid and solid phases, respectively but was absent in the deer. An OTU classified as *Geosporobacter* represented 1.2% of the sequences in both the liquid and solid phase in the deer but was <0.1% of the sequences in Elk. *Ruminococcus* was found in all animals at levels ranging from 1–2% in both the liquid and solid phases of digesta.

One of the most interesting observations was the high variability in the prevalence of *Fibrobacter.* A high percentage of the sequences in elk samples were classified as *Fibrobacter* however this phyla was found in very low levels in most of the deer samples. Comparing individual animals revealed high variability in the abundance of this genus. Within the elk samples, *Fibrobacter* was more abundant in rumen solids then rumen liquids (P = 1×10^−5^) ranging from 4.2% to 14.1% in the solid and 3.1% and 8.8% in the liquid phase of rumen contents. Within the white tailed deer samples one animal showed higher levels of *F. succinogenes* associated with the solid phase (0.8%).

### Comparison of bacterial communities between ruminants

**Figure 2:**
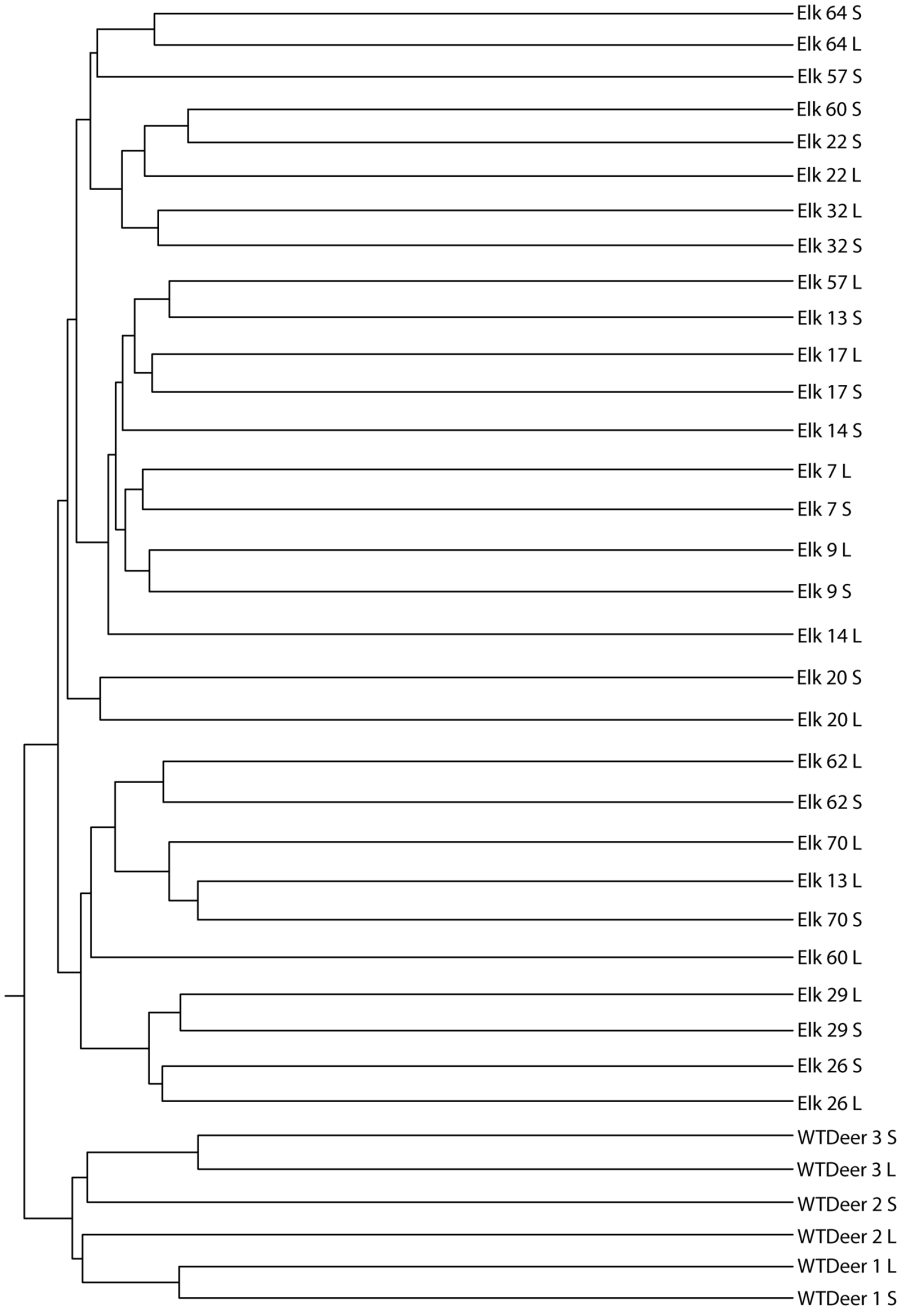
Microbial composition of wild ruminants assessed using Jaccard analysis of OTUs at 97% identity. Samples are labeled animal elk or white tailed deer - animal number - S (rumen solids) or L (rumen liquids).

Clustering of the samples based on a Jaccard similarity plot at 97% identity clearly showed that the deer samples clustered separately from the elk samples (Fig. 2). Furthermore, the samples generally clustered together based on the individual animal species and not on the basis of the digestive fraction (*i.e*. liquid versus solid). This is true of all the deer samples and 8 of the 15 elk samples. The elk samples form two large clades however there are no defining characteristics of these samples that explain this clustering. With the exception of samples 52, 53, 55, and 59, the elk samples did not cluster based on the location of the animal prior to harvest in southern Alberta and southwestern Saskatchewan.

**Figure 3:**
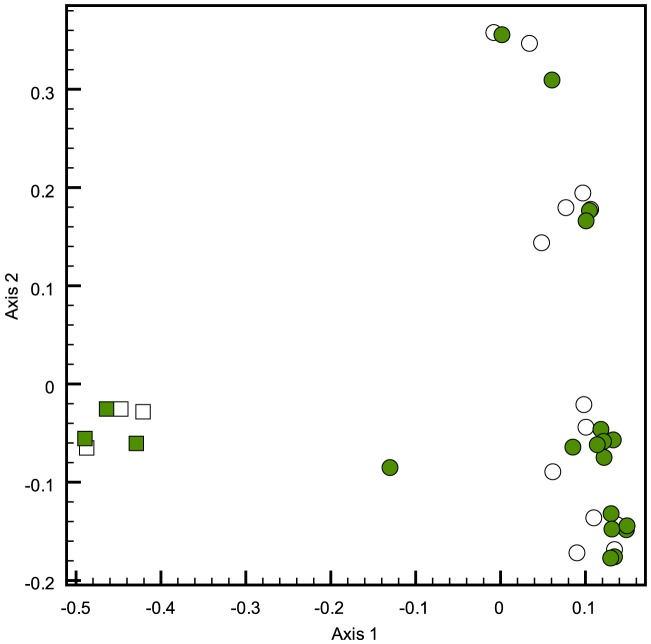
Principle component analysis of 16S profiles from rumen contents collected from elk (Solid-blue, Liquid-Black), white tailed deer (Solid-Red, Liquid-Green).

Weighted UniFrac analysis was carried out on the samples to quantify the differences in the microbial communities in the samples examined (Table 2). The microbial community associated with the white tail deer was significantly different (P<0.001) from the microbial communities associate with all elk samples. A comparison of the microbial communities in the liquid and solid rumen digesta showed that in some individuals the community varied significantly whereas in others it did not differ (Table 2). In particular, the liquid and solid phases in the deer sample did not show statistically significant differences in composition, however the difference in the Elk samples was significant. Principle component analysis showed distinct clustering of the communities based on ruminant species (Fig. 3). Elk samples showed a high degree of variability in community composition.

**Table 2:** Weighted-UniFrac comparison of microbial communities found in rumen samples based on Jaccard analysis of 97% similarity.

Samples compared	P value
WT deer Sol – WT deer Liq	0.53
WT deer Sol – Elk Sol	<0.001
WT deer Sol – Elk Liq	<0.001
WT deer Liq – Elk Sol	<0.001-0.42
WT deer Liq – Elk Liq	<0.001-0.42
Elk Sol – Elk Sol	<0.001-0.09
Elk Liq – Elk Liq	<0.001-0.30
Elk Sol – Elk Liq	<0.001-0.32

The P-value is a measure of the significance with which the microbial communities compared differ. P-values of <0.001 are highly significant, 0.001–0.01 are significant, 0.01–0.05 are marginally significant, 0.05–0.1 are suggestive and >0.1 is not significant. P-value ranges indicate the upper and lower limits of the calculation when multiple samples were compared.

### Individual variation in rumen bacteria

**Figure 4:**
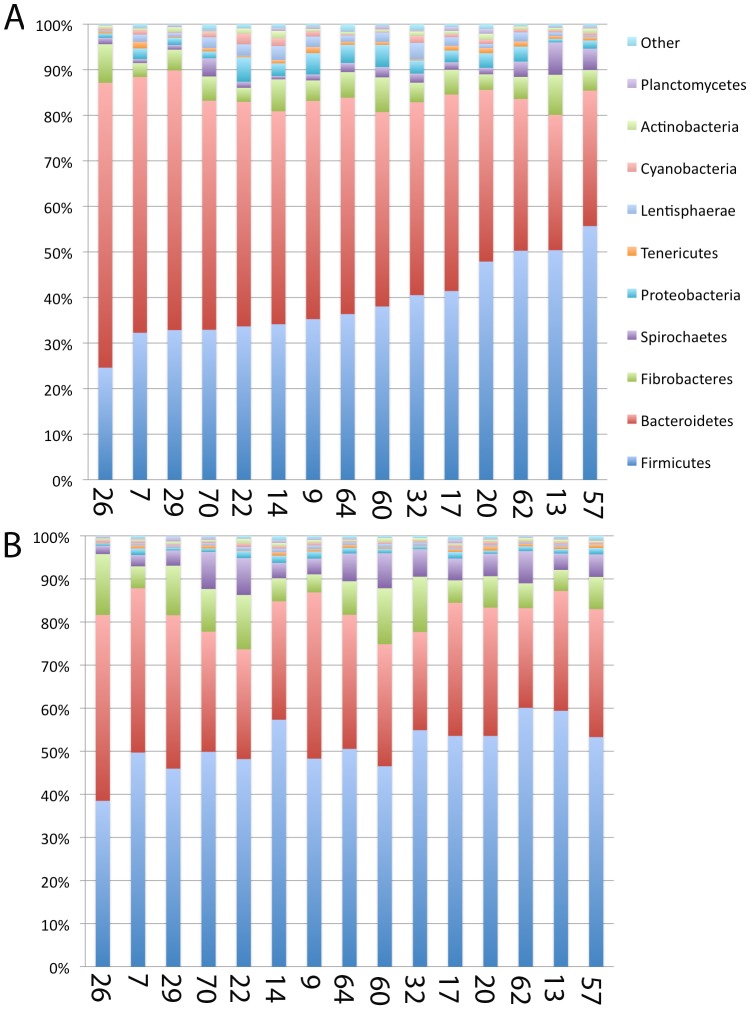
Diversity of bacterial phyla found in the Liquid (A) and Solid (B) fraction of rumen contents collected from 15 elk. Samples are arranged from the lowest to highest level of Firmicutes based on the percentage of total reads assigned to that phyla. Numbers along the x-axis indicate the animal identification number each sample was obtained from. The percentage of total reads (x-axis) assigned to each phylum is plotted for all 15 samples (y-axis).

Examination of samples from the elk provides a view of bacterial diversity within the same ruminant species over a larger number of individuals (n = 15). A comparison of the sequences classified at the phylum level showed that in all cases the majority of the bacteria belonged to Firmicutes and Bacteriodes (Fig. 4). In the elk samples, Firmicutes was found to be more abundant in the solid rumen contents (P = 1×10^−5^) whereas Bacteriodes was found at higher levels in the liquid fraction (P = 1×10^−5^). Fibrobacteres accounted for a large percentage of the sequences in elk with a higher percentage in the solid than the liquid fraction (P = 5×10^−3^). The Spirochaetes made up a larger fraction of the phyla in the solid phase, relative to the liquid phase (P = 3×10^−4^). Comparison of the distribution of bacterial phyla in all individuals revealed that in all cases Firmicutes, Bacteroidetes and Fibrobacteres were the dominant phyla, however the relative proportion was highly variable among individual hosts (Fig. 4). Deeper phylogenetic classification of the reads revealed that the majority of elk samples, both liquid and solid, were dominated by *Prevotella ruminocola*, an uncultured *Prevotella* species, RFN8-YE57 and *Fibrobacter succinogenes*. Although these genera tended to account for a large proportion of those identified, the relative amounts in each sample were highly variable. Animal 20 was unique in that it was the only individual that had significant levels of *Q. ovalis* and *Heliobacillus* within its bacterial community. These sequences were found at higher levels in the liquid phase of this animal.

### Core microbiome Analysis

The nature of the core microbiome was determined for all samples originating from white tailed deer and elk. Taxa that were found to be present in all of the samples in the analysis were deemed to be members of a core microbiome. The results of this analysis are shown in Table 3. There were 8 taxa from three phyla that were shared among all animals examined in this study. Analysis of the core microbiome of the elk rumen identified 12 conserved taxa, including *F. succinogenes*, the vadinHA42 cluster of *Ruminococcaceae, Treponema byrantii,* and an uncultured member of the genus *Saccharofermentans*. The core microbiome of the white tailed deer samples showed 11 shared taxa in the solid phase and 10 in the liquid phase, respectively, including *Q. ovalis* in the liquid and an uncultured member of the BS11 gut group in the solid fraction of digesta.

**Table 3:** Taxa identified within the core microbiome of the rumen of elk, white tailed deer.

All animals	Elk solid phase	Elk liquid phase	Deer solid phase	Deer liquid phase
*Lachnospiraceae* Wet75 sp1	*Lachnospiraceae* Wet75 sp1	*Lachnospiraceae* Wet75 sp1	*Lachnospiraceae* Wet75 sp1	*Lachnospiraceae* Wet75 sp1
Uncultured *Prevotella*	Uncultured *Prevotella*	Uncultured *Prevotella*	Uncultured *Prevotella*	Uncultured *Prevotella*
Unclassified Synergistetes	Unclassified Synergistetes	Unclassified Synergistetes	Unclassified Synergistetes	Unclassified Synergistetes
*Lachnospiraceae* Wet75 sp2	*Lachnospiraceae* Wet75 sp2	*Lachnospiraceae* Wet75 sp2	*Lachnospiraceae* Wet75 sp2	*Lachnospiraceae* Wet75 sp2
*Prevotella ruminicola*	*Prevotella Ruminicola*	*Prevotella ruminicola*	*Prevotella ruminicola*	*Prevotella ruminicola*
RFN8-YE57	RFN8-YE57	RFN8-YE57	RFN8-YE57	RFN8-YE57
Incertia sedis Clos-spor	Incertia sedis Clos-spor	Incertia sedis Clos-spor	Incertia sedis Clos-spor	Incertia sedis Clos-spor
RC9 gut group	RC9 gut group	RC9 gut group	RC9 gut group	RC9 gut group
	VadinHA42	VadinHA42	VadinHA42	*Quinella ovalis*
	*Treponema bryantii*	Uncultured *Para-prevotella*	Uncultured *Para-prevotella*	BS11 gut group
	*Fibrobacter succinogenes*	*Fibrobacter succinogenes*	BS11 gut group	*Prevotella byrantii*
	Uncultured *Saccharofermentans*	*Prevotella byrantii*	*Prevotella byrantii*	
**Phyla represented**				
Firmicutes, Bacteroidetes Synergistetes	Firmicutes, Bacteroidetes, Spirochaetes, Fibrobacteres Synergistetes	Firmicutes, Bacteroidetes, Fibrobacteres Synergistetes	Firmicutes, Bacteroidetes Synergistetes	Firmicutes, Bacteroidetes Synergistetes

## Discussion

Our results show that the bacterial communities found in the rumen of a number of wild ruminants are significantly different from each other. Furthermore, examination of multiple individuals shows that, although the phyla present within the rumen of a particular host species are generally conserved, the relative proportion of these microbes varies. These assertions are supported by distance-based clustering, using the Jaccard similarity index and principal component analysis, as well as weighted UniFrac calculations. We also compared the composition of the bacterial communities found within the liquid and solid phases of the rumen and found distinct differences in the composition of these communities in elk, but not deer. This agrees with the results of a comparison of the bacterial population in the liquid and solid rumen phases of cattle [5]. As is commonly seen in studies of the rumen microbiome, there was a high degree of variability in community composition between individuals. The deer samples were highly ruminated, having a thick, porridge-like consistency and were difficult to separate into liquid and solid fractions, which may contribute to the observed results.

A number of studies from various ruminants all point to Bacteroides and Firmicutes being the dominant phyla in the rumen ecosystem [5], [8], [9], [11], [14]. Our results confirm this finding and show that even within wild ruminants the above are the dominant phyla and compose the foundation of the core microbiome of wild ruminants. There have been relatively few studies that have used high throughput next-generation based sequencing to examine the bacterial diversity in the rumen thus far. One such study that examined the bovine rumen found *Prevotella* to be most prevalent followed by *Oscillibacter* and *Coprococcus* [13]. *Prevotella* and *Tannerella* were over represented in the liquid phase and *Butyrivibrio* and *Blautia* in the solid phase of digesta [13]. *Tannerella* was not observed in any of our samples and *Blautia* was seen only in low abundance (<0.3%) in elk. *Oscillibacter* was only found in white tailed deer and was more prevalent in the solid phase than the liquid phase of digesta (0.5% versus 0.1% of sequences). *Q. ovalis* was found to account for a substantial proportion of the rumen bacteria in white tailed deer but not elk. *Q. ovalis* is a member of the *Veillonellaceae* family that plays a role in the fermentation of starch and sugar to propionate, a characteristic consistent with its prevalence in the liquid phase of rumen contents. Another OTU that was found in high abundance is the rumen microbe labeled RFN8-YE57. This bacterium was first identified in the forestomach of an eastern grey kangaroo and belongs to the Family of the *Lachnospiracaeae*, within Clostridial cluster XIVa. It is related to *Butyrivibrio* and *Pseudobutyrivibrio,* which are non-sporulating, non-motile gram-negative rods that produce acetate, succinate, and N-butyrate as fermentation products [25]. Little is known about the specific role of this bacterium in the rumen, however the high abundance of this organism suggests it is an important member of the rumen microbiome. Another abundant OTU found in the elk samples but not in the deer was classified as *Victivallis*. The only described species, *Victivallis vadensis*, is a gram-negative, strictly anaerobic coccum, that displays cellobiose-degrading activity and is syntrophic with the methanogenic archaea *Methanospirillum hungatei* [26]. This genus was first identified in a human fecal sample but has not been previously described in the rumen. An OTU with similarity to the genus *Geosporobacter* was observed at levels of ∼1% of the sequences identified in deer. The only characterized member of this genus was isolated from a deep aquifer and is a sporulating, non-motile, gram-positive, obligate anaerobe that can ferment a diverse range of carbohydrates to generate acetate, H_2_ and CO_2_ [27].

*F. succinogenes* was highly abundant and a member of the core microbiome in the rumen of elk, but was virtually absent from the deer samples. This may be due to dietary differences and the fact that deer are browsers, as opposed to elk, which are grazers. The *Fibrobacter* sequences in elk were more abundant in the solid (8.5%) as compared to the liquid (5.4%) fraction (P = 5×10^−3^), which is consistent with the role of this organism in fiber degradation. A number of studies have found that *Fibrobacter* is not consistently found in the rumen [5], [10], [11], [15], [28], suggesting that the distribution of this organism may be quite variable in the rumen environment and that this organism only plays an important role in fiber digestion in some ruminant species. The high non-specific nuclease content of this bacterium [29], its sensitivity to lysis, as well as difficulties in amplifying *Fibrobacter* DNA [30] or natural variation in the levels of this organism may play roles in the observed variability of *Fibrobacter* in the rumen [31].

Two recent studies have utilized next-generation sequencing to investigate the bacterial diversity in Moose [15] and Reindeer [16]. Ishaq and Wright (2012) used the phylochip approach to examine the bacterial diversity in moose and found that the most predominate phyla were identified as Bacteriodetes and Proteobacteria. This contrasts other studies, which consistently show that Firmicutes and Bacteriodetes make up the dominant phyla in the rumen and is likely a result of both real differences in the individual rumen microbiome of the moose, and differences in the technique that was used in this study. A draw back of using this microarray-based approach is that species identification is restricted to the probes present on the chip. Furthermore, these probes were not specifically developed to represent bacterial members of the rumen environment, something acknowledged by the authors. The other studies examining the rumen microbiome of non-domesticated wild ruminants have focused on the Norwegian reindeer [11], [12], [16]. Both metagenomic sequencing targeting the V1-V3 of the 16s rRNA gene [16] and sequencing of full length 16s rRNA clones [11], [12] have been used and both have identified a number of novel sequences. These sequences were suggested to represent novel species that have not been observed in domesticated ruminants [11], [16]. Interestingly, the sequences observed in the reindeer microbial community were not observed in the samples examined in this study. These studies provide additional support to the hypothesis that the rumen microbiome varies with host species and that efforts should be made to study under-represented ruminant species.

## Conclusions

This work examined the bacterial communities found in elk and white tail deer. These hosts exhibit both similarities and differences to the rumen microbiome found in domesticated ruminants. Our results suggest that the current studies focusing primarily on domesticated bovine, sheep, and goats are not capturing the full diversity of microbes that are found within the rumen environment. A greater focus on examining the rumen microbiome of non-domesticated ruminants could help to identify novel microbes and enzymes of commercial interest.

## Supporting Information

Figure S1 **Representative rarefaction curves of wild ruminant samples.** Curves represent the number of OTUs at 97% similarity level observed as a function of sequencing depth. For clarity, not all of the samples examined are displayed.
(TIF)
