# Supplementary figures and images for: Diversity of Rumen Bacteria in Canadian Cervids
Source: PLoS One. 2014 Feb 27;9(2):e89682. doi: 10.1371/journal.pone.0089682 (PMC3937448; doi:10.1371/journal.pone.0089682)

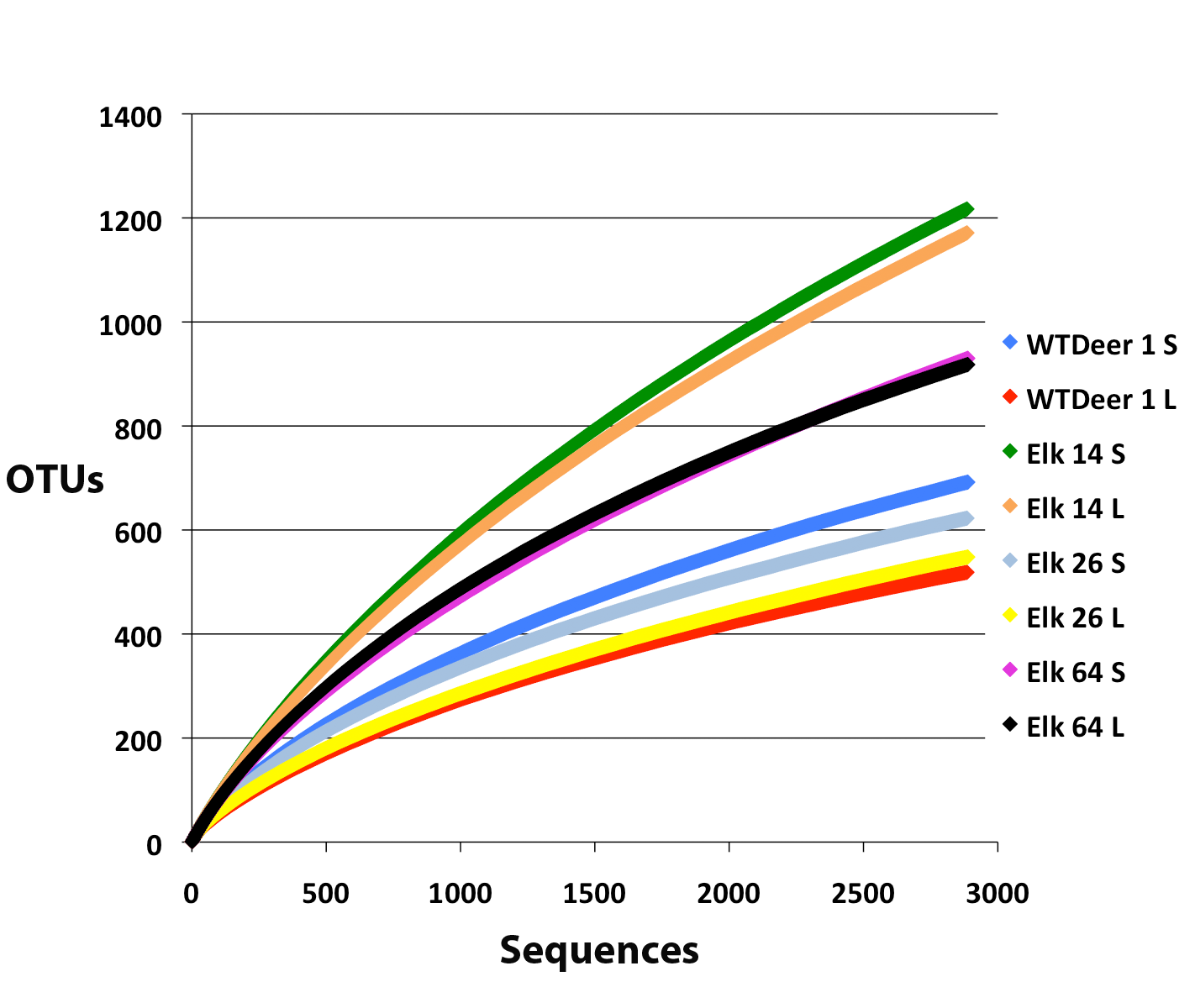

Supplement: Figure S1 — Representative rarefaction curves of wild ruminant samples. Curves represent the number of OTUs at 97% similarity level observed as a function of sequencing depth. For clarity, not all of the samples examined are displayed. (TIF) [file pone.0089682.s001.tif]
